# Neutrophil-to-lymphocyte ratio, platelet-to-lymphocyte ratio, and their dynamic changes during chemotherapy is useful to predict a more accurate prognosis of advanced biliary tract cancer

**DOI:** 10.18632/oncotarget.13731

**Published:** 2016-11-30

**Authors:** Kyoung-Min Cho, Hyunkyung Park, Do-Youn Oh, Tae-Yong Kim, Kyung-Hun Lee, Sae-Won Han, Seock-Ah Im, Tae-You Kim, Yung-Jue Bang

**Affiliations:** ^1^ Department of Internal Medicine, Seoul National University Hospital, Seoul National University College of Medicine, Seoul, Republic of Korea; ^2^ Cancer Research Institute, Seoul National University College of Medicine, Seoul, Republic of Korea; ^3^ Department of Internal Medicine, School of Medicine, Kyung Hee University, Seoul, Republic of Korea

**Keywords:** inflammation, prognosis, advanced biliary tract cancer, neutrophil-to-lymphocyte ratio

## Abstract

**Background and Purpose:**

Systemic inflammation is known to promote carcinogenesis in biliary tract cancer (BTC). Neutrophil-to-lymphocyte ratio (NLR) and platelet-to-lymphocyte ratio (PLR) are indicative of systemic inflammation. We evaluated the clinical significance of systemic inflammation measured by NLR and PLR in patients with advanced BTC. Additionally, we also co-analyzed the dynamics of NLR and PLR during chemotherapy.

**Methods:**

We reviewed 450 patients with unresectable BTC receiving palliative chemotherapy. NLR and PLR were obtained before initiation of palliative chemotherapy. Changes in NLR, PLR were obtained by subtracting the initial value from the value obtained after progression of chemotherapy.

**Results:**

Higher systemic inflammation status also had relation with a primary tumor site (p = 0.003) and higher levels of CEA (p = 0.038). The ROC cut-off values of NLR and PLR for predicting overall survival (OS) were 3.8 and 121, respectively. Patients with a high NLR or PLR had worse OS independently in multivariate analysis (6.90 vs. 9.80 months, p =0.002; 7.83 vs. 9.90 months, p =0.041, respectively). High NLR with increased NLR after chemotherapy is associated with worse OS and progression-free survival (PFS) (p < 0.001, p = 0.013 respectively). Results are similar for PLR.

**Conclusion:**

Systemic inflammation represented by NLR and PLR, predicts the OS of patients with advanced BTC who are receiving palliative chemotherapy. In addition, considering NLR/PLR with a dynamic change of NLR/PLR during chemotherapy might help to predict a more accurate prognosis.

## INTRODUCTION

Biliary tract cancers (BTCs) are tumors that are rare and have a poor prognosis. In Western countries, BTCs comprise less than 2% of all malignancies [1], but in Korea, they are more common, accounting for approximately 4% of malignancies [2]. Most patients who undergo surgical resection for BTC, generally experience a recurrence after a short disease-free period [3]. Overall survival (OS) in patients with advanced or metastatic BTC is less than 1 year [4]. The known prognostic factors include albumin, Eastern Cooperative Oncology Group (ECOG) Performance Status (PS), and serum carcinoembryonic antigen (CEA) level [5]; however, there is a lack of prognostic factors that can accurately predict the prognosis of BTC.

Chronic inflammation is known to promote carcinogenesis [6], which is triggered by cytokine or antitumor immunologic response within the tumor. Also, inflammation predisposes to cholangiocarcinoma [7]. A known risk factor for advanced BTC is chronic inflammation, which includes such things as gallstones, chronic hepatitis, primary sclerosing cholangitis, and liver fluke infection [8]. In gallbladder carcinoma, tumor necrosis factor-α (TNF-α) promotes lymph node metastasis through nuclear factor-κB-mediated [9]. In cholangiocarcinoma, the cytokine interleukin-6 (IL-6) is known to play a major role in tumor growth [10].

Although many biomarkers of systemic inflammation have recently been studied, excessive costs and technique factors prevent their clinical use. The most commonly investigated indicators of inflammation are those that are clinically most cost-effective and available, such as C-reactive protein (CRP) levels, platelet counts, and blood neutrophil counts [11]. Poor prognostic factors in various cancers include high neutrophil-to-lymphocyte ratio (NLR), high platelet-to-lymphocyte ratio (PLR), increased levels of neutrophils and platelets, and decreased levels of lymphocytes [12].

However, in BTC the role of NLR and PLR has rarely been analyzed. Only a few reports are available about NLR and PLR in patients with BTC who received curative surgery [13].

In the case of advanced BTC, little is known about the significance of NLR and PLR.

In this study, we investigated the prognostic role of NLR, PLR and CRP in patients with advanced BTC who received palliative chemotherapy in terms of response to chemotherapy, progression-free survival (PFS), and overall survival (OS). Additionally, we also co-analyzed the dynamics of NLR and PLR during chemotherapy.

## RESULTS

### Patients and treatments

**Figure 1:**
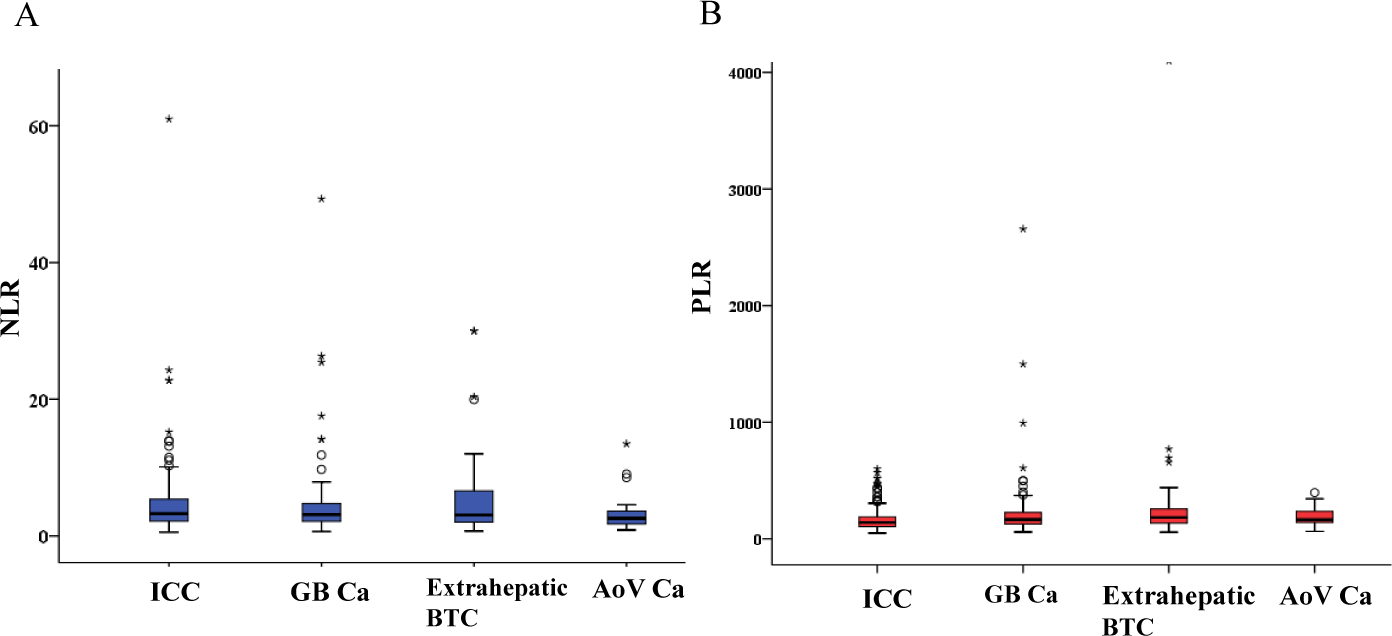
Distribution of NLR,PLR in each subtype of biliary tract cancers **A.** Distribution of NLR in each subtype of biliary tract cancers. **B.** Distribution of PLR in each subtype of biliary tract cancers.

A total of 450 patients with unresectable BTC were enrolled. Patient characteristics are shown in Table 1. The median age was 61 years (range, 26-84 years). The study cohort consisted of 295 (65.6%) males and 155 (34.4%) females. Malignancies included 214 (47.6%) intrahepatic cholangiocarcinomas (ICC), 151 (33.6%) gallbladder cancers (GB Ca), 54 (12.0%) extrahepatic bile duct cancers (Extrahepatic BTC), and 31 (6.9%) Ampulla of Vater cancers (AoV Ca). The Eastern Cooperative Oncology Group (ECOG) Performance Status (PS) was either 0 or 1 in 401(89.1 %) patients, and PS was 2 in 49 (10.9%) patients. The laboratory values at the time point of advanced BTC diagnosis were as follows: median CEA level, 2.8 ng/mL (range, 0.5-5410.0 ng/mL); cancer antigen 19-9(CA 19-9) value, 134.9 U/mL (range, 1.0-240,0000 U/mL); median total bilirubin, 0.9 mg/dL (range, 0.1-11.7,mg/dL); and albumin, 3.8 g/dL (range, 2.1-5.1 g/dL). The median value of NLR was 3.1 (range, 0.5-61.0), and the median value of PLR was 155.8 (range,48.6-4099.0). In terms of tumor origin, the median NLRs were 3.3, 3.1, 3.1, and 2.6 in ICC, GB Ca, Extrahepatic BTC, and AoV Ca, respectively. The median PLRs were 139.6, 166.4, 184.3, and 164.6 in ICC, GB Ca, Extrahapatic BTC, and AoV Ca, respectively (Figure 1). 204 patients (45.3%) received gemcitabine-based chemotherapy as a first-line treatment, and 232 patients (51.6 %) received fluoropyrimidine-based chemotherapy as first-line treatment.

**Table 1:** Baseline Characteristics of Patients

Characteristics	Patients (n=450)
Age — yr	
Median	61
Range	26-84
Sex-no (%)	
Male	295 (65.6%)
Female	155 (34.4%)
Primary tumor site — no. (%)	
ICC	214 (47.6%)
GB Ca	151 (33.6%)
Extrahepatic BTC	54 (12.0%)
AoV Ca	31 (6.9%)
ECOG PS— no. (%)	
0-1	401 (89.1%)
>2	49 (10.9%)
CEA (ng/ml) median (range)	2.8 (0.5-5410.0)
CA 19-9 (U/ml) median (range)	134.9 (1.0-2400000.0)
Total bilirubin (mg/dl) median (range)	0.9 (0.1-11.7)
Albumin (mg/dl) median (range)	3.8 (2.1-5.1)
1^st^ chemotherapy — no	
Gemcitabine -based	204 (45.3%)
FU-based	232 (51.6%)
Gemcitabine, FU -based	14 (3.1%)
NLR	
Median/ mean/ range	
All BTC	3.1/ 4.5/ 0.5-61.0
ICC	3.3/ 4.6/ 0.5-61.0
GB Ca	3.1/ 4.3/ 0.6-49.3
Extrahepatic BTC	3.1/ 5.7/ 0.7-30
AoV Ca	2.6/ 3.3/ 0.9-13.5
PLR	
Median, mean, range	
All BTC	155.8/ 200.4/ 48.6-4099.0
ICC	139.6/ 166.5/ 48.6-600.8
GB Ca	166.4/ 218.3/ 58.8-2657.4
Extrahepatic BTC	184.3/ 295.0/ 57.9-4099.0
AoV Ca	164.6/ 184.5/ 64.5-396.5
Serum CRP (mg/dl)	
Median, mean, range	2.5/ 4.4/ 0.0-25.7
All BTC	
ICC	2.4/ 4.4/ 0.0-24.9
GB Ca	1.8/ 4.3/ 0.0-25.7
Extrahepatic BTC	3.2/ 4.2/ 0.0-20.1
AoV Ca	4.0/ 5.4/ 0.2-16.3

Abbreviations: ICC, intrahepatic cholangiocarcinoma; BTC, biliary tract cancer; GB Ca, gallbladder cancer;AoV Ca, ampulla of Vater cancer; ECOG PS, Eastern Cooperative Oncology Group performance status;CEA, carcinoembryonic antigen; CA 19-9, cancer antigen 19-9; NLR, neutrophil-lymphocyte ratio; PLR, platelet-lymphocyte ratio; CRP, C-reactive protein

**Figure 2:**
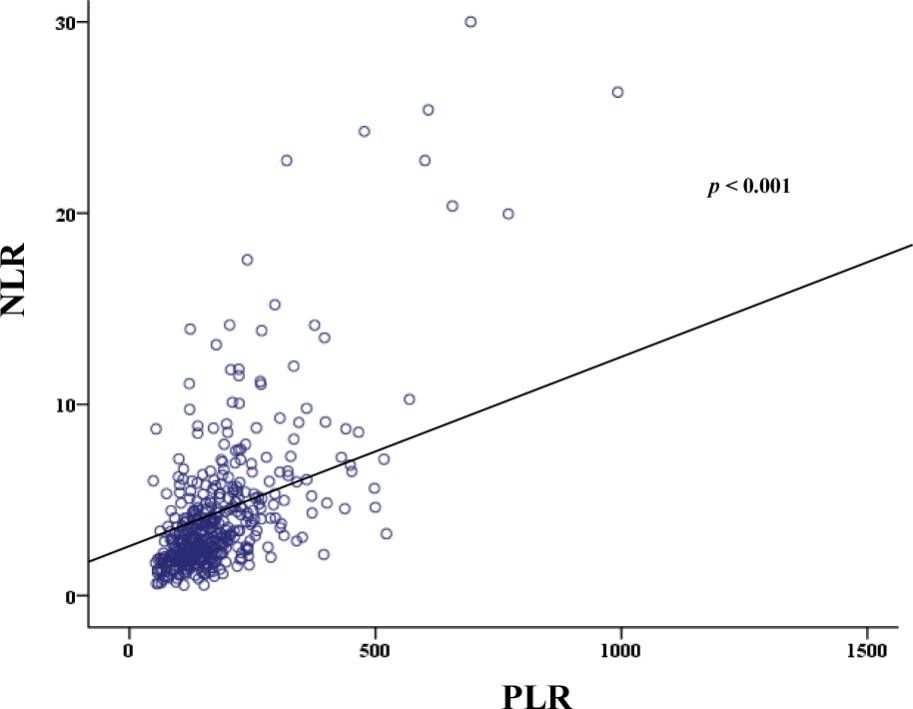
Correlation of NLR,PLR showing distribution of NLR,PLR

The area under the receiver operating characteristic (ROC) curve for NLR was 0.628 (95% CI: 0.573-0.683), and an NLR value of 3.8 was identified as the cut-off value for predicting OS, with a sensitivity of 52.3% and a specificity of 68.4%. The area under the ROC curve for PLR was 0.592 (95% CI: 0.536-0.648), and a PLR value of 121 was identified as the cut-off value for predicting OS, with a sensitivity of 78.4% and a specificity of 33.2%. Additionally, the area under the ROC curve for CRP was 0.552 (95% CI: 0.477-0.628), and an CRP value of 3.3 was identified as the cut-off value for predicting OS, with 49.1% sensitivity and 50.9% specificity. The patients were separated into 2 groups according to the cut-off values for NLR and PLR (low: <3.8 or high: ≥3.8; low: <121 or high ≥121, respectively). Our study showed a strong correlation for NLR and PLR (r=0.516, p < 0.001) (Figure 2).However, there was no correlation between NLR and CRP (p = 0.711) (Supplementary Figure S1A). Also, there was no correlation between PLR and CRP (p = 0.452) (Supplementary Figure S1B).

OS for all patients was 8.57 months (95% CI:7.90-9.24), and PFS was 4.20 months (95% CI: 3.75-4.70).

### Comparisons between high and low NLR groups

The patients with elevated CEA, low albumin and histologic poorly differentiation were more frequently found in the high NLR group (p = 0.038, p < 0.001, p = 0.029, respectively) (Table 2). In contrast, primary tumor site, the response rate, CRP were not different between high and low NLR groups (p =0.162, p =0.214, p = 0.683). Patients with high NLR showed worse PFS and OS compared with patients with low NLR (3.23 months vs 4.57 months, p = 0.028; 6.90 months vs 9.80 months, p < 0.001).

**Table 2:** Comparison between the high NLR and low NLR groups

	Low NLR group NLR <3.8 (N=262)	High NLR group NLR>3.8 (N=173)	*p *value
Age— yr			0.837
<65	173 (66.0%)	112 (64.7%)	
>65	89 (34.0%)	61 (35.3%)	
Sex, no (%)			**0.010**
Male	160 (61.1%)	127 (73.4%)	
Female	102 (38.9%)	46 (26.6%)	
ECOG Performance-Status			0.753
0-1	235 (89.7%)	153 (88.4%)	
>2	27 (10.3%)	20 (11.6%)	
Primary tumor site, no (%)			0.162
ICC	120 (45.8%)	88 (50.9%)	
GB Ca	89 (34.0%)	57 (32.9%)	
Extrahepatic BTC	30 (11.5%)	22 (12.7%)	
AoV Ca	23 (8.8%)	6 (3.5%)	
CEA (ng/mL), no (%)			**0.038**
CEA<5	155 (68.0%)	86 (57.3%)	
CEA> 5	73 (32.0%)	64 (42.7%)	
CA 19-9 (U/mL), no (%)			0.678
CA 19-9 <37	17 (6.5%)	15 (8.7%)	
CA 19-9 >37	77 (29.4%)	47 (27.2%)	
PLR, no (%)			0.082
PLR<121	98 (52.4%)	51 (42.1%)	
PLR>121	89 (47.6%)	70 (57.9%)	
Serum CRP (mg/dl), no (%)			0.683
<3.3	88 (57.5%)	52 (60.5%)	
>3.3	65 (42.5%)	34 (39.5%)	
Albumin (g/dl), no (%)			**< 0.001**
<3.3	40 (15.7%)	57 (33.9%)	
>3.3	215 (84.3%)	111 (66.1%)	
Metastatic site, no (%)			0.137
<2	218 (83.2%)	134 (77.5%)	
>2	44 (16.8%)	39 (22.5%)	
Histologic Differentiation			**0.029**
Well Differentiated	12 (9.7%)	3 (5.0%)	
Moderately Differentiated	69 (55.6%)	24 (40.0%)	
Poorly Differentiated	43 (34.7%)	33 (55.0%)	
1^st^line Chemotherapy ORR			0.214
PR or CR	46 (19.7%)	21 (14.3%)	
SD or PD	187 (80.3%)	126 (85.7%)	
PFS (Months)	4.57	3.23	**0.028**
	95% CI: 3.91-5.23	95% CI:2.20-4.27	
OS (Months)	9.80	6.90	**< 0.001**
	95% CI:8.97-10.64	95% CI:5.90-7.90	

Abbreviations: ICC, intrahepatic cholangiocarcinoma; BTC, biliary tract cancer; GB Ca, gallbladder cancer;AoV Ca, ampulla of Vater cancer; ECOG PS, Eastern Cooperative Oncology Group performance status;CEA, carcinoembryonic antigen; CA 19-9, cancer antigen 19-9; NLR, neutrophil-lymphocyte ratio; PLR, platelet-lymphocyte ratio; CRP, C-reactive protein; ORR , objective response rate; PR, partial response; CR, complete response; SD, stable disease; PD, progression disease; OS, overall survival; PFS, progression- free survival; 95% CI, 95% confidence interval

### Comparisons between high and low PLR groups

Unlike NLR, there were differences between low PLR group and high PLR group according to primary tumors (p = 0.003) (Table 3). Also, our study showed CEA, albumin, histologic differentiation had no differences between high and low PLR groups (p =0.075, p = 0.052, p =0.748, respectively). Patients with high PLR showed worse PFS and OS compared with patients with low PLR (4.10 months vs 4.73 months, p =0.091; 7.83 months vs 9.90 months, p = 0.012).

**Table 3:** Comparison between the high PLR and low PLR groups

	Low PLR group PLR<121 (N=119)	High PLR group PLR>121 (N=316)	*p *value
Age— yr			0.50
<65	75 (63.0%)	210 (66.5%)	
>65	44 (37.0%)	106 (33.5%)	
Sex, no (%)			0.055
Male	87 (73.1%)	200 (63.3%)	
Female	32 (26.9%)	116 (36.7%)	
ECOG Performance-Status			0.118
0-1	111 (93.3%)	277 (87.7%)	
>2	8 (6.7%)	39 (12.3%)	
Primary tumor site, no (%)			**0.003**
ICC	74 (62.2%)	133 (42.1%)	
GB Ca	28 (23.5%)	118 (37.3%)	
Extrahepatic BTC	11 (9.2%)	41 (13.0%)	
AoV Ca	6 (5.0%)	24 (7.6%)	
CEA (ng/mL), no (%)			0.075
CEA<5	103 (86.6%)	249 (78.8%)	
CEA> 5	16 (13.4%)	67 (21.2%)	
CA 19-9 (U/mL), no (%)			0.280
CA 19-9 <37	39 (34.8%)	85 (29.2%)	
CA 19-9 >37	73 (65.2%)	206 (70.8%)	
Serum CRP (mg/dl), no (%)			0.568
<3.3	39 (55.7%)	101 (59.8%)	
>3.3	31 (44.3%)	68 (40.2%)	
Albumin (g/dl), no (%)			0.052
<3.3	19 (16.4%)	78 (25.4%)	
>3.3	97 (83.6%)	229 (74.6%)	
Metastatic site, no (%)			0.075
<2	103 (86.6%)	249 (78.8%)	
>2	16 (13.4%)	67 (21.2%)	
Histologic Differentiation			0.748
Well Differentiated	3 (6.0%)	12 (9.0%)	
Moderately Differentiated	27 (54.0%)	66 (49.3%)	
Poorly Differentiated	20 (40.0%)	56 (41.8%)	
1^st^line Chemotherapy ORR			0.178
PR or CR	14 (13.1%)	53 (19.4%)	
SD or PD	93 (86.9%)	220 (80.6%)	
PFS (Months)	4.73	4.10	0.091
	95% CI:3.43-6.03	95% CI: 3.65-4.55	
OS (Months)	9.90	7.83	**0.012**
	95% CI 8.78-11.02	95% CI:7.10-8.57	

Abbreviations: ICC, intrahepatic cholangiocarcinoma; BTC, biliary tract cancer; GB Ca, gallbladder cancer;AoV Ca, ampulla of Vater cancer; ECOG PS, Eastern Cooperative Oncology Group performance status;CEA, carcinoembryonic antigen; CA 19-9, cancer antigen 19-9; PLR, platelet-lymphocyte ratio; CRP, C-reactive protein; ORR , objective response rate; PR, partial response; CR, complete response; SD, stable disease; PD, progression disease; OS, overall survival; PFS, progression- free survival; 95% CI, 95% confidence interv

### NLR, PLR, CPR and other prognostic factors for OS

**Figure 3:**
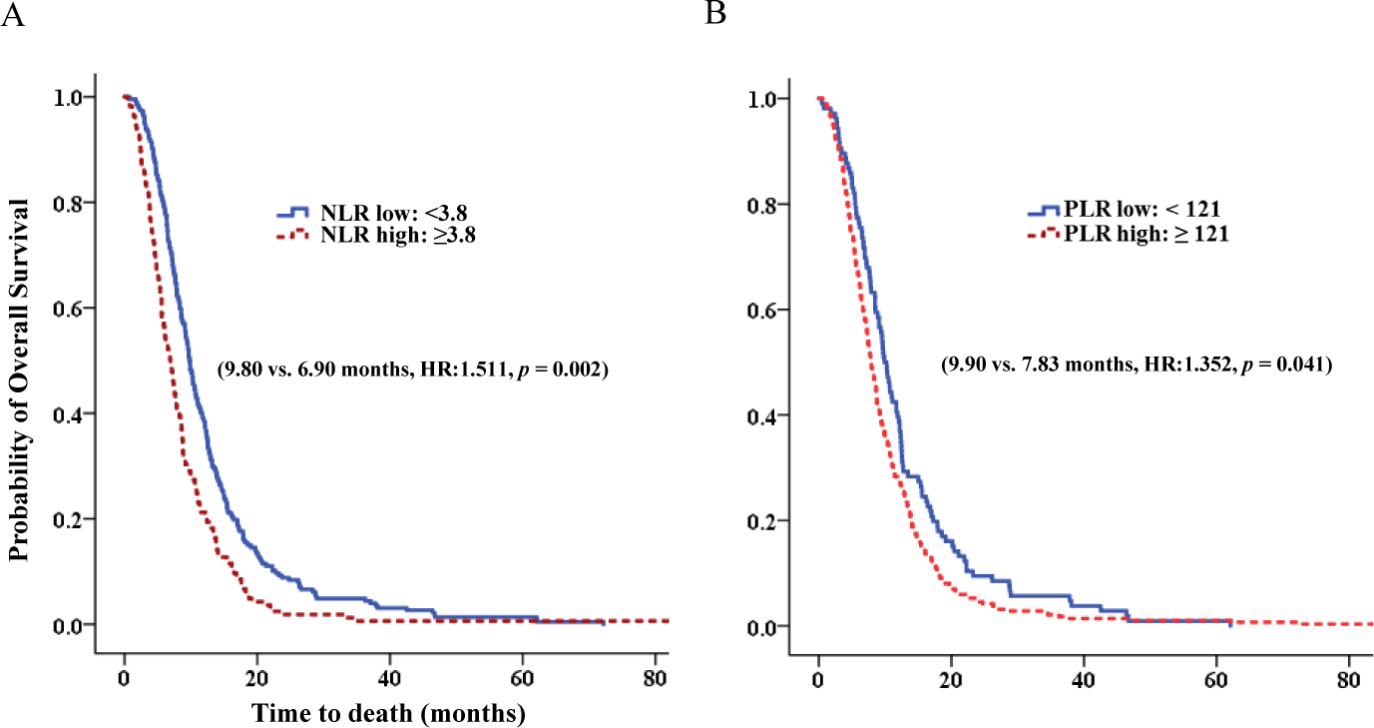
The overall survival according to NLR and PLR **A.** The overall survival according to NLR. **B.** The overall survival according to PLR.

In multivariate analysis, NLR ≥3.8 was a significant prognostic factor for OS (hazard ratio [HR]:1.511, 95% CI: 1.159-1.971, p = 0.002). PLR ≥121 was a significant prognostic factor for OS (HR:1.352, 95% CI: 1.013-1.803, p = 0.041) (Table 4, Figure 3). Age >65, CEA were independently associated with survival in advanced BTC. However, CRP ≥3.3, increased NLR after chemotherapy had no influence on OS (p = 0.162, p = 0.287). Whereas, increased PLR after chemotherapy significantly influenced on OS (HR: 1.721, 95% CI: 1.308-2.264, p < 0.001).

**Table 4:** Univariate & Multivariate Analysis for Overall Survival. OS (median: 8.57 month 95% CI:7.90-9.24)

Variable	Univariate analysis			Mutivariate		
	OS , months	95% CI	*p *value**	HR	95% CI	*p*^a^ value
Age — yr			**0.004**			**0.046**
<65	9.13	8.22-10.05		1		
>65	7.67	6.17-9.17		1.317	1.005-1.726	
Sex			0.709			
Male	8.57	7.64-9.49				
Female	8.63	7.74-9.53				
Primary Tumor Origin			0.244			
ICC	7.80	6.74-8.86				
GB Ca	9.20	8.00-10.40				
Extrahepatic BTC	8.57	7.39-9.75				
AoV Ca	11.47	7.08-15.85				
ECOG Performance Status			**< 0.001**			**0.012**
0-1	8.77	8.03-9.51		1		
>2	5.33	3.36-7.31		1.766	1.133-2.753	
CEA (ng/ml)			**< 0.001**			**0.001**
<5	9.50	8.50-10.50		1		
>5	7.23	6.19-8.28		1.679	1.232-2.288	
CA 19-9 (U/ml)			0.139			
<37	8.90	7.12-10.68				
>37	8.63	7.86-9.41				
Albumin (g/dl)			**0.022**			0.185
<3.3	6.90	5.73-8.07		1		
>3.3	8.9	8.13-9.67		0.821	0.613-1.099	
Serum CRP (mg/dl)			0.162			
<3.3	9.50	7.95-11.05				
>3.3	7.60	6.67-8.54				
NLR			**< 0.001**			**0.002**
NLR <3.8	9.80	8.97-10.64		1		
NLR >3.8	6.90	5.90-7.90		1.511	1.159-1.971	
PLR			**0.012**			**0.041**
PLR <121	9.90	8.78-11.02		1		
PLR >121	7.83	7.10-8.57		1.352	1.013-1.803	
Change in NLR			0.287			
Maintained	9.93	8.89-10.10				
Increased	7.67	6.08-9.25				
Change in PLR			**0.002**			**< 0.001**
Maintained	9.93	8.49-11.38		1		
Increased	7.77	6.16-9.37		1.721	1.308-2.264	

Abbreviations: ICC, intrahepatic cholangiocarcinoma; BTC, biliary tract cancer; GB Ca, gallbladder cancer;AoV Ca, ampulla of Vater cancer; ECOG PS, Eastern Cooperative Oncology Group performance status;CEA, carcinoembryonic antigen; CA 19-9, cancer antigen 19-9; NLR, neutrophil-lymphocyte ratio; PLR, platelet-lymphocyte ratio; CRP, C-reactive protein; OS, overall survival;; 95% CI, 95% confidence interval

^†^Change in NLR,PLR : maintained ≥ -10% , decreased < -10%

### NLR, PLR, and other prognostic factors for PFS

**Figure 4:**
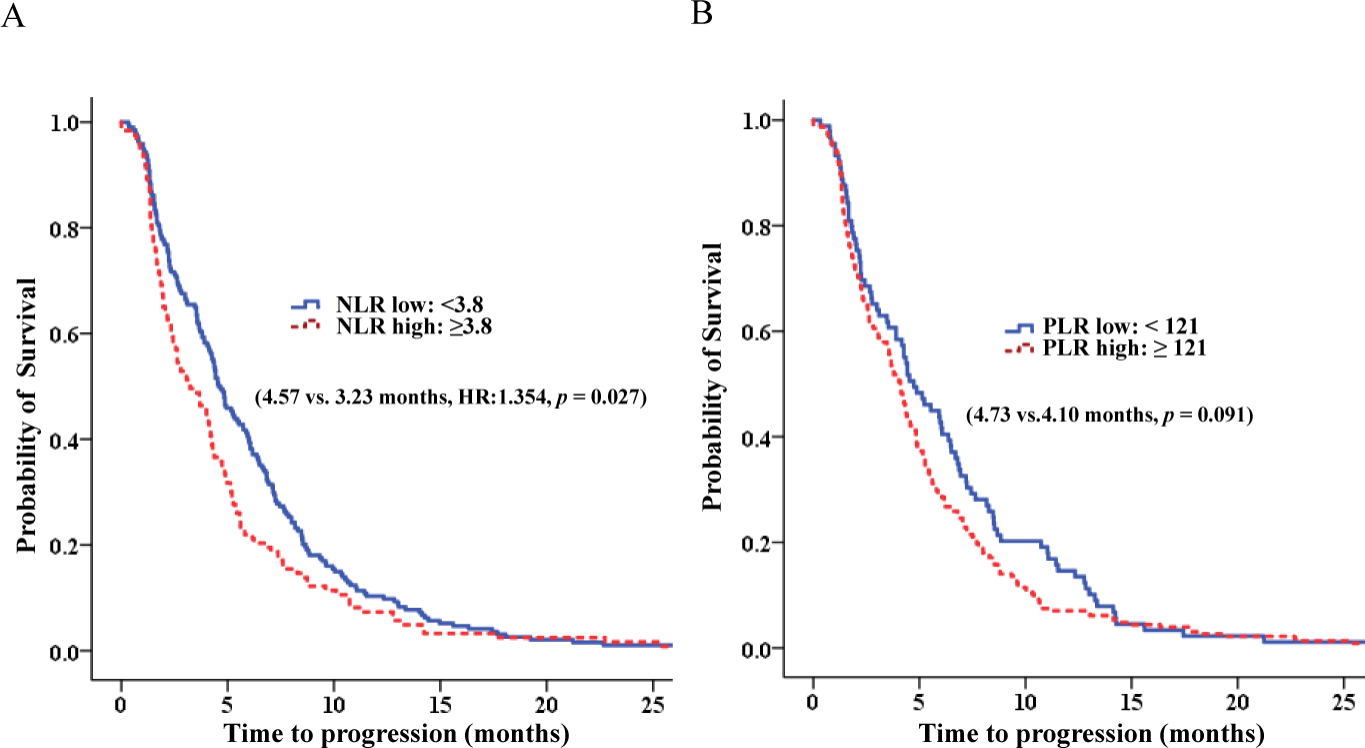
The progression free survival according to NLR and PLR **A.** The progression free survival according to NLR. **B.** The progression free survival according to PLR

In multivariate analysis, a high NLR together with an increased PLR after chemotherapy, CEA and CA 19-9 levels, and response rate were significant prognostic factors for PFS (Table 5, Figure 4).

**Table 5:** Univariate & Multivariate Analysis for PFS. PFS (median: 4.20 month 95% CI:3.75-4.65)

Variable	Univariate analysis			Mutivariate		
	PFS, months	95% CI	*p* value	HR	95% CI	*p^a^* value
Age — yr			0.504			
<65	4.20	3.59-4.82				
>65	4.23	3.49-4.98				
Sex			0.768			
Male	4.20	3.72-4.58				
Female	4.27	3.34-5.19				
Primary Tumor Origin			0.398			
ICC	3.70	2.66-4.74				
GB Ca	4.53	3.92-5.15				
Extrahepatic BTC	4.83	3.69-5.98				
AoV Ca	4.43	1.33-7.53				
ECOG Performance Status			0.056			
0-1	4.27	3.81-4.72				
>2	1.97	1.41-2.53				
CEA (ng/ml)			**0.042**			**< 0.001**
<5	4.43	3.99-4.88		1		
>5	4.13	2.85-5.42		1.782	1.302-2.439	
CA 19-9 (U/ml)			**0.013**			**0.008**
<37	4.53	3.80-5.27		1		
>37	4.27	3.78-4.75		1.432	1.100-1.863	
Albumin (g/dl)			**0.019**			0.051
<3.3	3.60	2.31-4.89		1		
>3.3	4.37	3.88-4.86		0.734	0.539-1.001	
CRP			0.824			
<3.3	3.67	2.58-4.75				
>3.3	3.80	2.51-5.10				
NLR			**0.028**			**0.027**
NLR <3.8	4.57	3.91-5.23		1		
NLR >3.8	3.23	2.20-4.27		1.354	1.035-1.772	
PLR			0.091			
PLR <121	4.73	3.43-6. 03				
PLR >121	4.10	3.65-4.56				
Change in NLR			0.688			
Maintained	4.20	3.73-4.67				
Increased	3.80	2.71-4.88				
Change in PLR			**0.010**			**0.009**
Maintained	4.43	3.97-4.90		1		
Increased	3.10	2.06-4.15		1.415	1.089-1.839	
ORR			**< 0.001**			**< 0.001**
PR or CR	8.17	7.24-9.10		1		
SD or SD	3.53	2.80-4.27		2.596	1.874-3.596	

Abbreviations: ICC, intrahepatic cholangiocarcinoma; BTC, biliary tract cancer; GB Ca, gallbladder cancer;AoV Ca, ampulla of Vater cancer; ECOG PS, Eastern Cooperative Oncology Group performance status;CEA, carcinoembryonic antigen; CA 19-9, cancer antigen 19-9; NLR, neutrophil-lymphocyte ratio; PLR, platelet-lymphocyte ratio; CRP, C-reactive protein; ORR , objective response rate; PR, partial response; CR, complete response; SD, stable disease; PD, progression disease; OS, overall survival; PFS, progression- free survival; 95% CI, 95% confidence interval

^†^Change in NLR,PLR : maintained ≥ -10% , decreased < -10%

### Analysis of NLR and PLR with co-analysis of dynamics of NLR and PLR during chemotherapy

**Figure 5:**
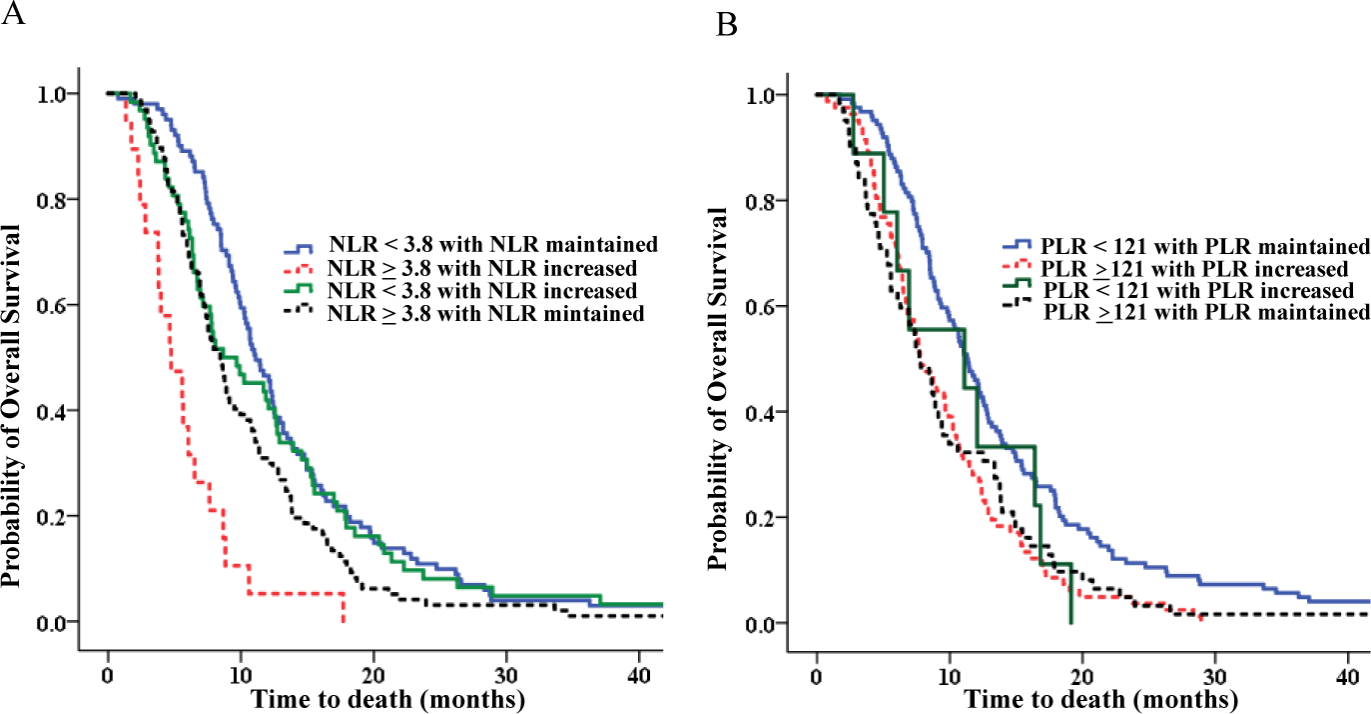
OS analysis of NLR and PLR with co-analysis of dynamics of NLR and PLR **A.** OS analysis of NLR with co-analysis of dynamics of NLR. **B.** OS analysis of PLR with co-analysis of dynamics of PLR.

**Figure 6:**
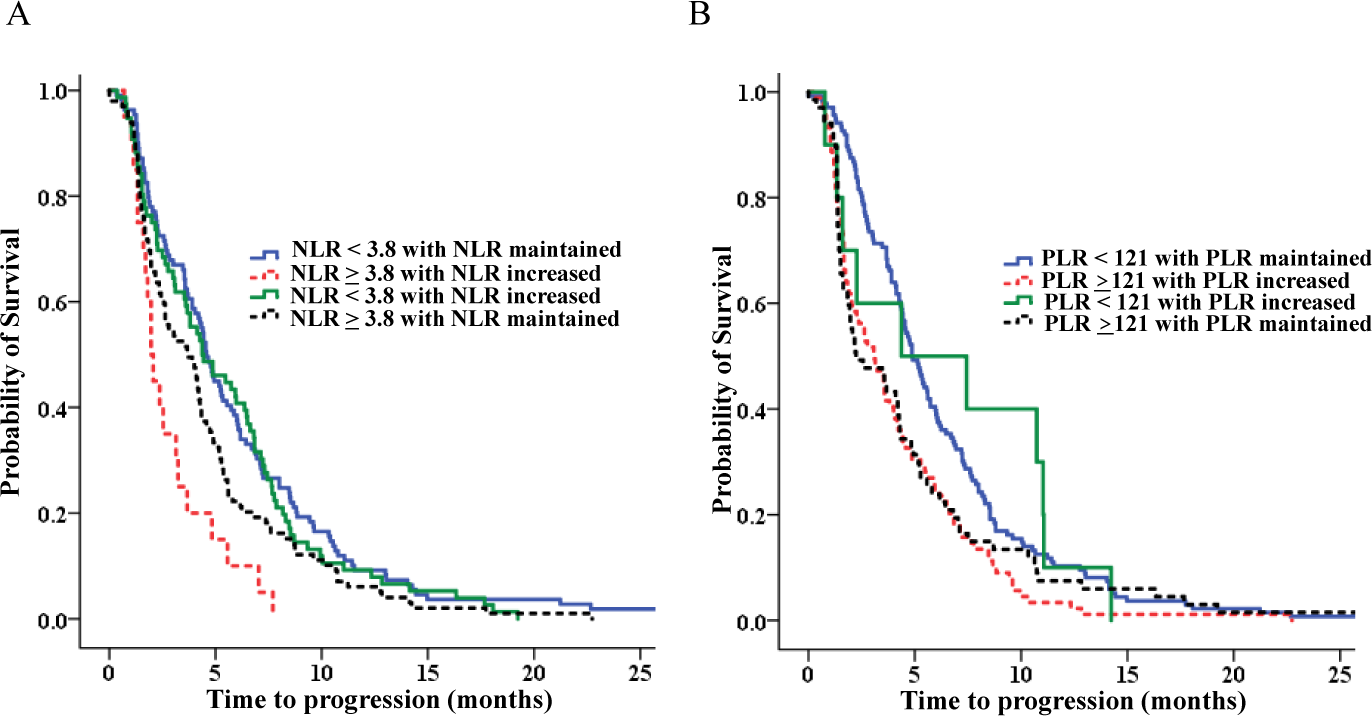
PFS analysis of NLR and PLR with co-analysis of dynamics of NLR and PLR **A.** PFS analysis of NLR with co-analysis of dynamics of NLR. **B.** PFS analysis of PLR with co-analysis of dynamics of PLR.

Analysis of NLR in patients in whom NLR changes had occurred (low NLR with maintained NLR, low NLR with increased NLR, high NLR with maintained NLR, high NLR with increased NLR) revealed that patients with high NLR with increased NLR had the shortest survival (p <0.001; Table 6). Compared with a median OS of 11.10 months for patients with low NLR with maintained NLR, patients with high NLR with increased NLR had the worst OS (median OS 4.73 months; HR 3.113; p < 0.001; Table 6, Figure 5). In addition, compared with a median PFS of 4.57 months for patients with low NLR with maintained NLR, patients with high NLR with increased NLR had the worst PFS (median OS 1.97 months; HR 2.151; p = 0.007; Table 7, Figure 6). Analyses according to PLR showed similar results.

**Table 6:** OS analysis of NLR and PLR with co-analysis of dynamics of NLR and PLR during chemotherapy.

Group	Change	Univariate analysis			Mutivariate		
		OS , months	95% CI	*p* value**	HR	95% CI	*p *^a ^value**
Low NLR group	Maintained	11.10	9.71-12.50	**< 0.001**	1		**< 0.001**
NLR<3.8	Increased	8.67	4.87-12.46		0.992	0.708-1.390	0.962
High NLR group	Maintained	8.43	7.39-9.48		1.399	1.036-1.889	**0.028**
NLR >3.8	Increased	4.73	2.46-7.01		3.113	1.826-5.306	**< 0.001**
Low PLR group	Maintained	11.30	9.90-12.70	**0.001**	1		**0.002**
PLR <121	Increased	7.13	0.67-13.59		1.471	0.750-2.887	0.262
High PLR group	Maintained	7.70	6.03-9.37		1.363	0.983-1.891	0.064
PLR >121	Increased	7.76	6.26-9.28		1.786	1.321-2.413	**< 0.001**

Abbreviations: NLR, neutrophil-lymphocyte ratio; PLR, platelet-lymphocyte ratio

^†^Change in NLR,PLR : maintained ≥ -10% , decreased < -10%

*aP* values were calculated using the Cox-proportional hazard model, age, PS,CEA,albumin,

**Table 7:** PFS analysis of NLR and PLR with co-analysis of dynamics of NLR and PLR during chemotherapy.

		Univariate analysis			Mutivariate		
		PFS , months	95% CI	*p *value**	HR	95% CI	*p*^a^ value
Low NLR group	Maintained	4.57	4.01-5.13	**0.001**	1		**0.013**
NLR <3.8	Increased3	4.37	2.59-6.15		0.967	0.689-1.358	0.848
High NLR group	Maintained4	3.70	2.49-4.91		1.426	1.037-1.963	**0.029**
NLR >3.8	Increased2	1.97	1.58-2.36		2.151	1.236-3.744	**0.007**
Low PLR group	Maintained	4.87	4.19-5.55	**0.003**	1		**0.007**
PLR <121	Increased3	4.37	0.00-10.66		0.831	0.381-1.812	0.641
High PLR group	Maintained4	2.23	0.40-4.07		1.231	0.890-1.703	0.209
PLR >121	Increased2	3.10	2.12-4.08		1.725	1.249-2.381	**0.001**

Abbreviations: NLR, neutrophil-lymphocyte ratio; PLR, platelet-lymphocyte ratio

^†^Change in NLR,PLR : maintained ≥ -10% , decreased < -10%

*aP* values were calculated using the Cox-proportional hazard model, PS, albumin,CEA,CA 19-9, ORR.

## DISCUSSION

In this study, we found that systemic inflammation represented by NLR and PLR predicts the overall survival of patients with unresectable BTC who are receiving palliative chemotherapy.

Tumor metastasis depends on interactions between tumor cells and the host microenvironment. This involves blood cells, components of the coagulation system, stromal cells, and the extracellular matrix. Cells within the bloodstream include endothelial cells, platelets, lymphocytes, macrophages, mast cells, and fibroblasts [14]. These cells produce cytokines, which are cytotoxic mediators like reactive oxygen species (ROS), tumor necrosis factor-alpha (TNF-α), and interleukins, leading to tumor progression [15]. High neutrophil counts have long been reported to negatively affect the prognosis of various other cancers [11]. The neutrophils release ROS, which causes point mutations and DNA damage [16]. Also, neutrophils release neutrophil elastase, which is related to tumor cell proliferation, VEGF-related angiogenesis [17]. Through these mechanisms, neutrophils promote proliferation, invasion, and angiogenesis in cancer. Furthermore, T cells in tumors induce an immune response to the lesion, which includes a high number of cytotoxic T lymphocytes and increased neoplastic cell apoptosis [18]. Lymphocytopenia suppresses the immune response due to a marked decrease in T4 helper lymphocytes and an increase in T8 suppressor lymphocytes [19].

Platelet count is an additional index of systemic inflammation caused by the tumor. Platelet adhesion and aggregation leads to the formation and release of platelet granules that contain active proteases, growth factors, matrix proteins, and chemokines that enhance tumor progression [20]. Also, platelets release many factors, such as angiopoietin 1, epidermal growth factor, basic fibroblast growth factor, and interleukin-1β and IL-8 cytokines, which regulate the angiogenic process [21].

Therefore, NLR and PLR are considered to be the balance between inflammatory status and antitumor status. Patients with elevated NLR and PLR have relative lymphocytopenia, neutrophil leukocytosis, and thrombocytosis, which cause pro-tumor inflammation. Our study showed that NLR is associated with the serum albumin level. Scheede-Bergdahl et al. have reported an association between cancer cachexia and interleukin (IL)-1b levels [22], and Tisdale et al. have also reported that cachexia is associated with cytokines, including tumor necrosis factor-a (TNF-a), IL-1, IL-2, and IL-6, and interferon-r (IFN-r). In light of these findings, NLR could be used as a predictive marker of cancer cachexia. NLR is also significantly associated with CEA, which is an indicator of tumor burden [23]. This suggests that the greater the tumor burden, the greater the NLR, and this is consistent with reports that there is a significant association between higher NLR, larger tumor size, and the extent of micro-vascular invasion in cases of resectable BTC [24]. In addition, NLR is significantly associated with tumor cell differentiation.

In contrast with NLR, PLR varies in BTC according to tumor origin (p = 0.003). Compared with other primary tumors, ampulla of Vater cancer (AoV Ca) has a greater distribution of lower PLR values. Thus, differences in overall survival (OS) according to the primary tumor in BTC might be associated to some extent with PLR. PLR also had a slight association with CEA, CA 19-9, and albumin levels and with tumor cell differentiation. Considering the above, when we look at both NLR and PLR, we can more accurately predict the prognosis of BTC.

In a previous report about NLR as a prognostic factor in advanced BTC [25], an association was reported between NLR and OS, but no association was found between NLR and PFS. In our study, an association was found between NLR OS and PFS. Interestingly, in our study, no relationship existed between changes in NLR or PLR. However, when we analyzed subgroups according to NLR and dynamic change in NRL during chemotherapy, our study reveals that high NLR with increased NLR after chemotherapy was associcated with a worse survival outcome (Table 6). There was a 6.4 month OS difference between high NLR with increased NLR group and low NLR with maintained NLR group. The results for PLR are similar. Furthermore, our study reveals that high NLR with increased NLR after chemotherapy is associcated with a worse PFS. So, in light of the above information, considering NLR and PLR along with change might help to more accurately predict heterogeneous prognoses in BTC.

Currently, no consensus exists regarding NLR and PLR cut-off values, which vary across tumor types [12, 26]. In the present study, NLR and PLR cut-off values of 3.8 and 121, respectively, were selected using ROC curves, and the same procedure was used as reported in other studies [26]. McNamara et al. demonstrated a median NLR cut-off value of 3 as a prognostic marker in advanced BTC [25]. Dumitrascu et al. demonstrated a median NLR cut-off value of 3.3 as a prognostic marker in resectable BTC [13].

Recently, there have been several reports showing that circulating tumor cells are associated with prognosis [23, 27], and in our previous published reports, the soluble form of programmed death-ligand 1 (PD-L1) in peripheral blood was associated with prognosis in advanced BTC [28]. Further studies to find the association of NLR with circulating stem cells might be worthy.

A limitation of the present study is its retrospective design. Another caveat is that NLR is known to be a nonspecific marker of inflammation. Therefore, the presence of another systemic disease could have influenced the NLR value.

In conclusion, systemic inflammation represented by NLR and PLR predicts the OS of patients with advanced BTC who are receiving palliative chemotherapy. In addition, considering NLR and PLR with dynamic changes of NLR and PLR during chemotherapy might help to predict a more accurate prognosis in advanced BTC.

## PATIENTS AND METHODS

### Study population and data collection

We consecutively enrolled patients with advanced BTC who were diagnosed at the Seoul National University Hospital (Seoul, South Korea) between 2003 and 2013. All patients fulfilled the following criteria: (1) Histologically confirmed biliary tract cancer, (2) Unresectable, metastatic biliary tract cancer, and (3) Had received at least one cycle of palliative chemotherapy, (4) The recurrent biliary tract cancer patients were excluded.

The medical records of each patient were reviewed with respect to laboratory complete blood count (CBC) at the time of diagnosis, sex, age, tumor origin, localization, tumor marker, PFS, and OS.

The NLR was defined as the absolute neutrophil count in peripheral blood divided by the absolute lymphocyte count. The PLR was defined as the absolute platelet count in peripheral blood divided by the absolute lymphocyte count. Changes in NLR, PLR were obtained by subtracting the initial value from the value obtained after progression of chemotherapy.

### Statistics

A receiver operator characteristic (ROC) curve was used to assess the best discriminative cut-off values of NLR, PLR, and CRP while showing an association between NLR, PLR, and OS. The chi-square test was used to compare baseline characteristics of patients between the NLR and PLR groups. Overall survival (OS) was calculated from the first day of diagnosis of advanced BTC to the date of death, and progression-free survival (PFS) was calculated from the first day of palliative chemotherapy to the date of progression or any cause of death. We analyzed the OS of all patients in this study. The OS and PFS were assessed using Kaplan-Meier estimates. The log-rank test was used to assess the equality of the survivor function across groups. The Cox proportional hazards model with 95% confidence interval was used for the multivariate analysis to assess the effect of patient characteristics and other significant prognostic factors such as NLR and PLR. A p value of 0.05 or less was considered statistically significant. All analyses were performed using SPSS software for Windows (version 21; IBM SPSS, Somers, NY).

#### Ethics

The study protocol was reviewed and approved by the Institutional Review Board of Seoul National University Hospital (IRB No.H-1306-109-500). All studies were conducted according to guidelines (Declaration of Helsinki) for biomedical research.
